# Correlation and risk factor analysis of multifidus muscle atrophy in degenerative lumbar spondylolisthesis

**DOI:** 10.3389/fmed.2025.1609660

**Published:** 2025-06-11

**Authors:** Cong Zhang, Rui Sun, Xiaotao Wu, Xiaozhi Sun

**Affiliations:** ^1^Department of Spine Surgery, Zhongda Hospital, School of Medicine, Southeast University, Nanjing, China; ^2^Surgery Research Center, School of Medicine, Southeast University, Nanjing, China; ^3^Department of Orthopedic Trauma, Yizheng Hospital of Traditional Chinese Medicine, Yizheng, China

**Keywords:** degenerative lumbar spondylolisthesis, osteoporosis, multifidus muscle atrophy, paraspinal muscles, fat infiltration

## Abstract

**Objective:**

We evaluated differences in multifidus muscle atrophy (MMA) among patients with degenerative lumbar spondylolisthesis (DLS) across various segments and grades of spondylolisthesis, analysed the correlation between DLS and MMA, identified risk factors contributing to MMA, and provided a clinical reference for the prevention and treatment of MMA.

**Methods:**

This retrospective study analysed data from 213 patients diagnosed with single-segment DLS between September 2020 and January 2022. Participants were categorised into three groups based on the affected spinal segment: L3 (*n* = 27), L4 (*n* = 140), and L5 (*n* = 46). The LCSA/GCSA ratio was calculated to assess the extent of MMA. Differences in MMA and its correlation with DLS severity were analysed across different spondylolisthesis grades. Furthermore, based on the Kjaer classification, patients were stratified into Mild and Severe MMA groups. A multivariate logistic regression analysis was performed to identify risk factors influencing the degree of MMA in DLS patients.

**Results:**

The LCSA/GCSA ratio at the spondylolisthesis segment was significantly lower than that at the non-spondylolisthesis segment (*p* < 0.001). When comparing LCSA/GCSA ratios between different grades of lumbar spondylolisthesis (Grade I and II), no statistically significant differences were observed (*p* > 0.05). In the general population, a strong positive correlation was identified between the degree of MMA and the VAS and ODI scores for low back pain, whereas no significant correlation was found with the VAS score for leg pain. Age, BMI, and osteoporosis demonstrated statistically significant differences between the two groups (*p* < 0.05). Multivariate logistic regression analysis identified age, BMI, and osteoporosis as significant risk factors for MMA progression in DLS patients (*p* < 0.05).

**Conclusion:**

DLS patients exhibit MMA, with more pronounced atrophy at the spondylolisthesis-affected segment. Age, BMI, and osteoporosis are independent risk factors for MMA progression in DLS patients. Clinically, it is crucial to identify and monitor high-risk patients with these factors and implement early preventive and therapeutic interventions to mitigate disease progression.

## 1 Introduction

Degenerative lumbar spondylolisthesis (DLS) refers to the anterior displacement of the upper vertebral body relative to the lower vertebral body during lumbar degeneration. Unlike isthmic spondylolisthesis, DLS is characterised by an intact lumbar isthmus. The primary clinical manifestations include low back pain radiating to the leg due to nerve root compression. DLS is a prevalent degenerative condition of the lumbar spine, with a higher incidence in women than in men (1). Numerous studies have investigated the pathogenesis of DLS, and the widely accepted hypothesis attributes its development to lumbar disc degeneration, facet joint degeneration, and laxity of the surrounding muscles and ligaments (2–4). These mechanisms primarily emphasise the role of intervertebral joint instability in the progression of DLS. However, the overall stability and flexibility of the lumbar spine depend on multiple anatomical structures, and current theories do not fully elucidate the aetiology of DLS. The paraspinal muscles of the lumbar spine primarily include the multifidus, longissimus, iliopsoas, and psoas major muscles. Among these, the multifidus muscle is the most critical component, playing a key role in resisting rotational and translational movements of the spine. Its primary physiological function is to maintain the physiological lordosis of the lumbar spine, thereby contributing to spinal stability (5, 6).

The multifidus muscle consists of multiple independent muscle bundles, with superficial bundles spanning several vertebral bodies in a directional manner. Conversely, the deep muscle bundles are segmentally arranged, overlapping one another, and positioned close to the rotational centre of the lumbar facet joints. These structural characteristics allow even slight contractions of the multifidus muscle to generate significant motor force (7). The muscle fibres of the multifidus are rich in proprioceptors, particularly muscle spindles, which are highly sensitive to tensile stimuli and changes in muscle length. When the body changes position, the muscle spindles in the multifidus detect tension and generate nerve impulses, which are transmitted to the spinal cord, triggering regulatory signals. This activation prompts the multifidus muscle to contract, counteracting rotational movements and vertebral slippage, contributing to lumbar spine stabilisation (8, 9).

The primary manifestations of multifidus muscle degeneration include a reduction in muscle mass and increased fat infiltration (10, 11). Multifidus muscle mass gradually declines with age, decreasing by approximately 1% per year after the age of 40 (12, 13). Due to its unique anatomical and biomechanical properties, progressive degeneration and fat infiltration of the multifidus muscle impair its autonomous contractile function, ultimately compromising segmental stability (14). Currently, studies on the correlation between multifidus muscle atrophy (MMA) and DLS remain limited and controversial. We analysed the relationship between DLS and MMA through lumbar magnetic resonance imaging (MRI) measurements. In addition, the severity of MMA in patients will be classified into Mild and Severe groups based on the Kjaer classification (15). A retrospective analysis will be conducted to identify risk factors for MMA in DLS, providing insights for early and effective interventions to mitigate MMA and prevent the development of DLS in clinical practice.

## 2 Data and methods

### 2.1 Research subjects

A retrospective study was conducted on inpatients diagnosed with single-segment DLS who were admitted to the Department of Spine Surgery at Southeast University Affiliated Zhongda Hospital between September 2020 and January 2022. Patients aged > 45 years who were diagnosed with single-segment DLS and had complete imaging data, including lumbar spine MRI, computed tomography, anteroposterior and lateral radiographs, and dynamic radiographs, were included. Patients with isthmic spondylolisthesis, ankylosing spondylitis, rheumatoid arthritis, congenital lumbar spine malformations, a history of lumbar spine surgery, primary or metastatic tumours involving the lumbar spine, or lumbar spine fractures were excluded. We also reduced potential bias by applying strict inclusion and exclusion criteria (such as excluding patients with chronic liver disease, chronic kidney disease, or those receiving acupuncture or massage therapy, as these conditions and treatments may affect MMA). Additionally, a comparison of baseline data for sex, age, BMI, osteoporosis, hypertension, and diabetes among the three groups of DLS patients showed no statistically significant differences, indicating that the groups are comparable.

During this retrospective study, strict inclusion and exclusion criteria were applied, and dedicated personnel were responsible for follow-up and recording clinical data, thereby minimising data selection bias and enhancing the accuracy of retrospective data recording. In addition, 20 healthy older adult volunteers were recruited as a control group. These individuals exhibited no history of low back or leg pain, lumbar spinal pathology, trauma, or prior lumbar spine surgery. The control group was matched with the DLS group in terms of age, sex, and BMI. The study was approved by the Ethical Committee of the Zhongda Hospital, Southeast University (No. 2019ZDSYLL101-P01). Informed written consent was obtained from all patients.

### 2.2 Methods and evaluation

#### 2.2.1 Recording baseline patient information

Patient demographic and clinical data were recorded, including sex, age, body mass index (BMI), presence of osteoporosis, hypertension, and diabetes mellitus, the level of lumbar spondylolisthesis, and relevant laboratory examination findings.

Patients underwent anteroposterior, lateral, and dynamic lumbar spine radiographs. Spondylolisthesis severity was assessed using the Meyerding classification system based on lateral radiographs, categorising patients into Grade I or II (16). Patients were stratified into three groups based on the affected vertebral segment: the L3 group (*n* = 27; 24 Grade I, 3 Grade II), the L4 group (*n* = 140; 126 Grade I, 14 Grade II), and the L5 group (*n* = 46; 40 Grade I, 6 Grade II).

#### 2.2.2 Measurement of MMA

MRI offers high sensitivity for detecting early degeneration of paravertebral muscles and provides superior visualisation of surrounding muscles, adipose tissue, and neural structures without radiation exposure. This modality is widely recognised as an effective method for quantifying muscle atrophy and fat infiltration (11). Data were collected from 20 older adult healthy volunteers to serve as a control group. All patients with spondylolisthesis and control subjects underwent standardised scanning using a 3.0 T Siemens MRI scanner (Siemens Healthcare, Erlangen, Germany). T2-weighted images were obtained at the L3/4, L4/5, and L5/S1 intervertebral disc levels, corresponding to the maximal cross-sectional area of the paravertebral muscles (17). MRI scans parameters during image acquisition are as follows, slice thickness: 4 mm, FOV: 200 mm, pixel matrix: 512*512, Voxel: 0.65*0.49*4 mm.

**Figure 1:**
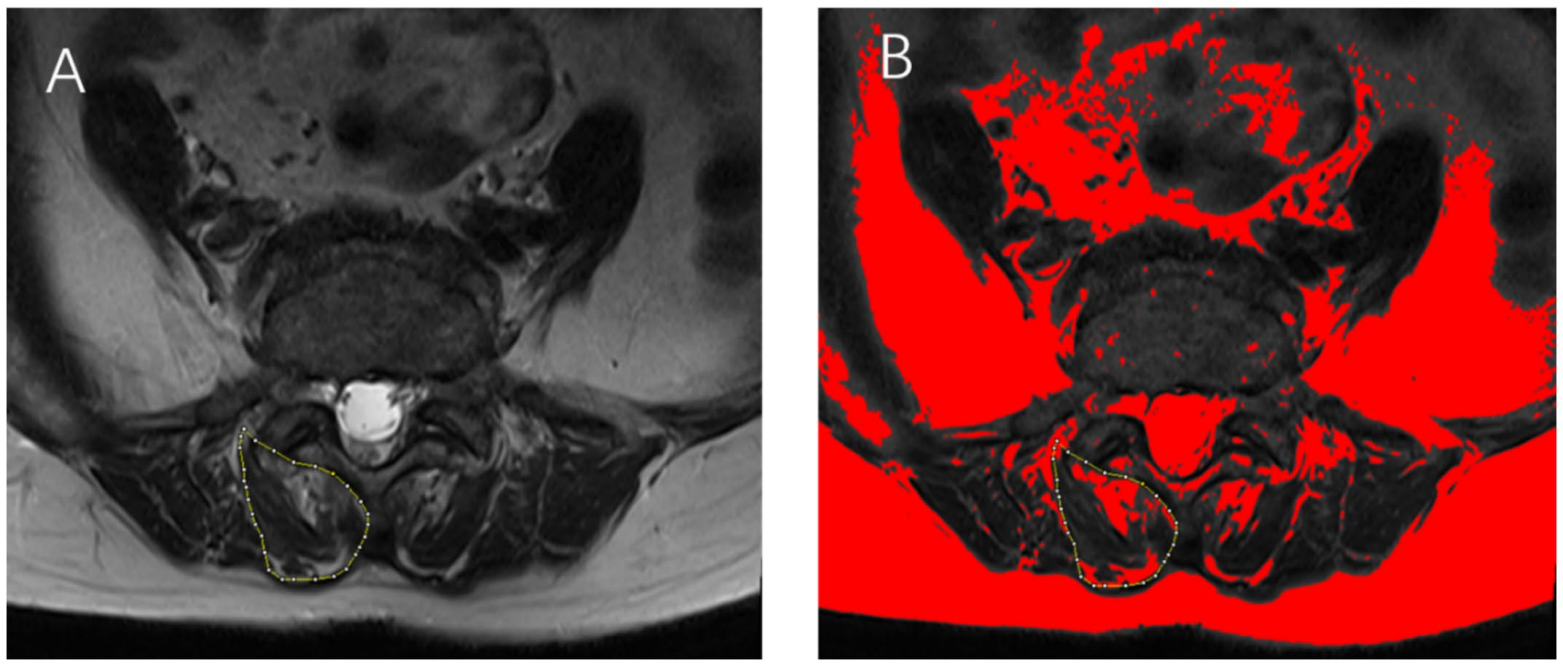
**(A)** Axial T2-weighted magnetic resonance imaging (MRI) of a 65-year-old female patient, illustrating the multifidus muscle area at the L4/5 level, outlined in yellow. **(B)** The fat infiltration area within the multifidus muscle is highlighted in red. The total multifidus muscle area is calculated by summing the measured areas on both sides of the lumbar spine.

The cross-sectional area of the multifidus muscle on both sides of the lumbar spine was measured at the specified levels using ImageJ software (National Institutes of Health, Bethesda, MD, United States) and summed to determine the gross cross-sectional area (GCSA). Similarly, the lean cross-sectional area (LCSA) of the multifidus muscle on both sides was measured and summed to represent the total LCSA (Figure 1). Click on the threshold button of Image J software and adjust it until the fat infiltration area in the image is completely covered. The degree of MMA was calculated as the ratio of LCSA to GCSA (LCSA/GCSA), with a higher ratio indicating milder atrophy and a lower ratio indicating more severe atrophy (18). All measurements were independently conducted by two experienced radiologists. In cases of discrepancies, consensus was reached through discussion. Both radiologists had extensive clinical experience and received standardised training on measurement protocols prior to the study. Both radiologists received our standardised training and were knowledgeable in how to control grayscale values, adjust thresholds, and identify tissue edges. In addition, they were blinded to the research objectives, to minimise potential bias.

#### 2.2.3 Visual analogue scale and Oswestry disability index scores

Preoperative VAS and ODI scores were obtained for all three patient groups. The.

VAS score ranges from 0 to 10, where 0 indicates no pain and 10 represents extreme pain, with higher scores reflecting greater pain severity. The ODI questionnaire is used to assess functional disability due to low back pain, with the score calculated by dividing the actual score by the maximum possible score and multiplying by 100%. A higher ODI score indicates more severe functional impairment related to low back pain.

#### 2.2.4 Risk factor analysis

According to the Kjaer classification, patients were categorised into Mild and Severe groups based on the degree of MMA. Differences in age, sex, BMI, osteoporosis, laboratory examination indicators, and other relevant factors between the two groups were compared. In addition, a multifactor logistic regression analysis was conducted to identify the risk factors influencing the degree of MMA in DLS patients.

### 2.3 Statistical analyses

Statistical analyses were performed using SPSS version 20.0 (IBM Corp., Armonk, NY, United States). Continuous variables are presented as mean ± standard deviation, whereas categorical variables are expressed as frequencies and percentages. Independent samples t-tests and chi-square (χ^2^) tests were used for comparisons between two groups. In this study, the L3 group included a total of 27 patients. Exact tests were used for comparisons between two groups (e.g., L3 group vs. L4 group, L3 group vs. L5 group). One-way analysis of variance (ANOVA) was used for comparisons among multiple groups. Multiple linear regression analysis was conducted to compare differences in LCSA/GCSA at different lumbar levels between the spondylolisthesis and control groups. Pearson correlation analysis was used to assess the correlation of MMA between the spondylolisthesis and non-spondylolisthesis segments. In addition, a multifactor logistic regression analysis was performed to identify risk factors influencing the degree of MMA in DLS patients. A significance level of *α* = 0.05 was set, with *p* < 0.05 considered statistically significant.

## 3 Results

### 3.1 Demographic characteristics of DLS patients

Our study included 213 patients with single-segment DLS and 20 healthy older adult individuals as a control group. All spondylolisthesis patients had single-stage DLS with complete imaging data.

The mean age of the DLS patients was 63.04 ± 9.25 (45–91) years. Among the 213 participants, 56 were male (26.3%), and 157 were female (73.7%). The distribution of spondylolisthesis levels was as follows: L3 segment (*n* = 27; 24 Grade I, 3 Grade II), including 6 males and 21 females, aged 46 to 83 years; L4 segment (*n* = 140; 126 Grade I, 14 Grade II), including 35 males and 105 females, aged 47 to 88 years; and L5 segment (*n* = 46; 40 Grade I, 6 Grade II), including 15 males and 31 females, aged 45–91 years. Comparison of demographic variables, including sex, age, BMI, presence of osteoporosis, hypertension, and diabetes mellitus, among the three spondylolisthesis groups revealed no statistically significant differences (*p* > 0.05), indicating baseline comparability (Table 1).

**Table 1:** Comparison of the general information of the three groups of patients with DLS.

Variables	L3 group	L4 group	L5 group	*F*/*χ*^2^	*p*
	*n* = 27	*n* = 140	*n* = 46		
Age	61.52 ± 9.91	64.07 ± 9.09	60.80 ± 9.02	2.619	0.075
Male, n(%)	6 (22.22)	35 (25.00)	15 (32.61)	1.298	0.522
BMI (kg/m^2^)	24.50 ± 2.93	25.01 ± 3.18	25.26 ± 2.80	0.510	0.600
Osteoporosis, n(%)	12 (48.15)	73 (52.14)	22 (47.83)	0.673	0.714
Hypertension, n(%)	8 (29.63)	68 (48.57)	21 (45.65)	3.275	0.194
Diabetes mellitus, n(%)	2 (7.41)	27 (19.29)	4 (8.70)	4.510	0.105

SD, standard deviation; BMI, body mass index; DLS, degenerative lumbar spondylolisthesis.

### 3.2 Comparison of MMA between DLS and control groups

After matching for age, sex, and BMI, multiple linear regression analysis revealed that the LCSA/GCSA ratio of the multifidus muscle at the L3/4, L4/5, and L5/S1 levels in all three spondylolisthesis groups was significantly lower than that in the control group, with statistically significant differences (*p* < 0.001; Table 2).

**Table 2:** Comparison of the degree of MMA at three intervertebral disc levels between DLS patients and the control group.

Groups	Level of intervertebral discs	DLS patients	Control group	*t*	*p*
L3	L3/4	49.29 ± 6.59	78.91 ± 4.78	˗17.183	< 0.001
	L4/5	65.78 ± 8.71	77.29 ± 5.86	˗4.936	< 0.001
	L5/S1	63.74 ± 10.57	76.34 ± 4.00	˗4.869	< 0.001
L4	L3/4	63.13 ± 9.46	78.91 ± 4.78	˗6.569	< 0.001
	L4/5	49.30 ± 11.84	77.29 ± 5.86	˗9.575	< 0.001
	L5/S1	63.09 ± 9.74	76.34 ± 4.00	˗5.175	< 0.001
L5	L3/4	66.54 ± 8.19	78.91 ± 4.78	˗6.176	< 0.001
	L4/5	61.82 ± 8.51	77.29 ± 5.86	˗7.274	< 0.001
	L5/S1	52.30 ± 11.46	76.34 ± 4.00	˗9.245	< 0.001

DLS, degenerative lumbar spondylolisthesis; MMA, multifidus muscle atrophy.

### 3.3 Comparison of MMA at different intervertebral disc levels in DLS patients

**Figure 2:**
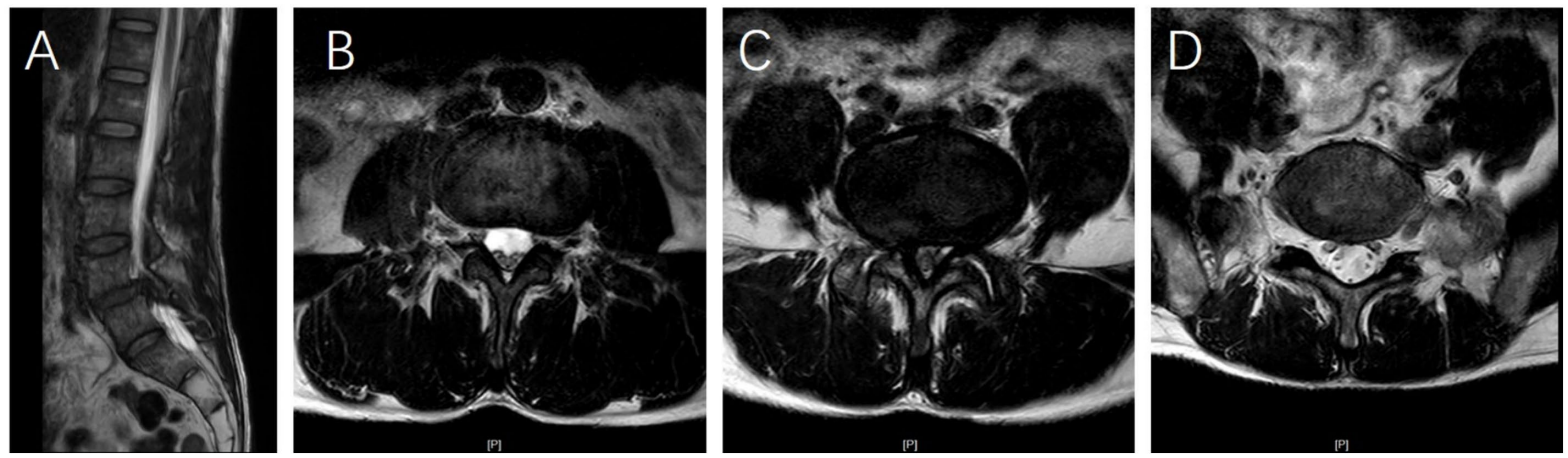
**(A)** Sagittal T2-weighted magnetic resonance imaging (MRI) of a 62-year-old female patient demonstrating L4 vertebral spondylolisthesis. **(B–D)** Axial T2-weighted MRI scans at the L3/4, L4/5, and L5/S1 levels reveal that multifidus muscle atrophy (MMA) is most severe at the L4/5 level compared to the L3/4 and L5/S1 levels, whereas the degree of MMA is similar between the L3/4 and L5/S1 levels.

A comparison of multifidus LCSA/GCSA between the spondylolisthesis and non-spondylolisthesis segments in DLS patients revealed a statistically significant difference (*p* < 0.001; Table 3). In contrast, no statistically significant differences were observed in multifidus LCSA/GCSA in the L3/4, L4/5, L5/S1 segments in the control group (*p* > 0.05; Table 3; Figure 2).

**Table 3:** Comparison of the degree of MMA at different intervertebral disc levels between DLS patients and the control group.

Groups	L3/4 level	L4/5 level	L5/S1 level	*F*	*p-*values
L3	49.29 ± 6.59	65.78 ± 8.71	63.74 ± 10.57	28.310	< 0.001
L4	63.13 ± 9.46	49.30 ± 11.84	63.09 ± 9.74	82.179	< 0.001
L5	66.54 ± 8.19	61.82 ± 8.51	52.30 ± 11.46	26.812	< 0.001
Controls	78.91 ± 4.78	77.29 ± 5.86	76.34 ± 4.00	1.384	0.259

DLS, degenerative lumbar spondylolisthesis; MMA, multifidus muscle atrophy.

### 3.4 Comparison of MMA in DLS patients with grade I and II

According to the Meyerding grading system, DLS patients are divided into Grade I and Grade II. No statistically significant differences were observed in the LCSA/GCSA of the multifidus muscle between patients with different grades of spondylolisthesis at the same segment (*p* > 0.05; Table 4).

**Table 4:** Comparison of LCSA/GCSA between different grades of DLS at the same segment.

Groups	Level of intervertebral discs	Grade I	Grade II	*t*	*p-*values
		*n* = 190	*n* = 23		
L3	L3/4	49.16 ± 6.14	50.39 ± 11.40	˗0.300	0.767
	L4/5	65.52 ± 8.58	67.87 ± 11.51	˗0.433	0.668
	L5/S1	63.18 ± 10.80	68.18 ± 8.72	˗0.766	0.451
L4	L3/4	62.88 ± 9.34	65.45 ± 10.56	˗0.990	0.337
	L4/5	49.09 ± 11.33	51.28 ± 16.17	0.010	0.629
	L5/S1	63.12 ± 9.29	62.77 ± 13.62	0.326	0.897
L5	L3/4	67.02 ± 8.21	63.34 ± 8.00	0.837	0.311
	L4/5	62.57 ± 7.79	56.80 ± 11.98	0.500	0.123
	L5/S1	53.04 ± 11.38	47.31 ± 11.78	1.532	0.258

LCSA/GCSA, lean cross-sectional area to gross cross-sectional area ratio; DLS, degenerative lumbar spondylolisthesis.

### 3.5 Comparison of the correlation between multifidus LCSA/GCSA in the spondylolisthesis and non-spondylolisthesis segments

**Figure 3:**
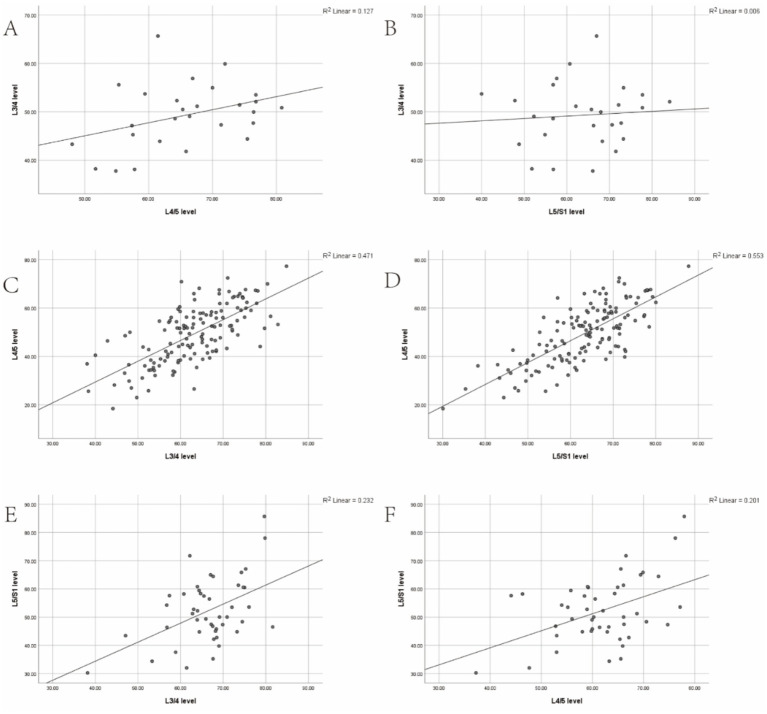
**(A,B)** Correlation between the degree of multifidus muscle atrophy (MMA) at the L3/4 level and the degree of MMA at the L4/5 and L5/S1 levels in the L3 group. **(C,D)** Correlation between the degree of MMA at the L4/5 level and the degree of MMA at the L3/4 and L5/S1 levels in the L3 group. **(E,F)** Correlation between the degree of MMA at the L5/S1 level and the degree of MMA at the L3/4 and L4/5 levels in the L3 group.

In the L3 group, a weak positive correlation was observed between the L3/4 level and the L4/5 and L5/S1 levels (*r* = 0.357, *p* > 0.05; *r* = 0.078, *p* > 0.05). In the L4 group, a strong positive correlation was found between the L4/5 level and the L3/4 and L5/S1 levels (*r* = 0.686, *p* < 0.001; *r* = 0.744, *p* < 0.001). In the L5 group, a moderate positive correlation was identified between the L5/S1 level and the L3/4 and L4/5 levels (*r* = 0.482, *p* < 0.001; *r* = 0.448, *p* < 0.05; Table 5; Figure 3).

**Table 5:** Comparison of the correlation between multifidus LCSA/GCSA in the spondylolisthesis and non-spondylolisthesis segments.

Groups	L3/4 level	L4/5 level	L5/S1 level
L3	1.000	0.357	0.078
L4	0.686^**^	1.000	0.744^**^
L5	0.482^**^	0.448^**^	1.000

LCSA/GCSA, lean cross-sectional area to gross cross-sectional area ratio. **indicates *p* < 0.001.

Scatter plots were constructed to illustrate the correlation trends of LCSA/GCSA between the spondylolisthesis and non-spondylolisthesis segments of the multifidus muscle in three groups, clearly depicting the strength of these correlations.

### 3.6 Correlation analysis of the degree of MMA with VAS scores for low back and leg pain and ODI scores

A significant negative correlation was observed between the degree of MMA and the VAS and ODI scores for low back pain across the three patient groups (*r* = ˗0.907, *p* < 0.001; *r* = ˗0.407, *p* < 0.05; *r* = ˗0.942, *p* < 0.001; *r* = ˗0.541, *p* < 0.001; *r* = ˗0.856, *p* < 0.001; *r* = ˗0.298, *p* < 0.05). However, no significant correlation was observed between MMA degree and the VAS score for leg pain (*r* = ˗0.078, *p* > 0.05; *r* = ˗0.045, *p* > 0.05; *r* = ˗0.124, *p* > 0.05). Similarly, in the general population, a significant negative correlation was observed between the degree of MMA and the VAS and ODI scores for low back pain (*r* = ˗0.927, *p* < 0.001; *r* = ˗0.474, *p* < 0.001), whereas no significant correlation was found with the VAS score for leg pain (*r* = ˗0.117, *p* > 0.05; Table 6).

**Table 6:** Correlation analysis of the degree of MMA with preoperative VAS scores for low back and leg pain and ODI scores.

Variables	L3 (*n* = 27)		L4 (*n* = 140)		L5 (*n* = 46)		General population (*n* = 213)	
	*r*	*p-*values	*r*	*p-*values	*r*	*p-*values	R	*p-*values
Low back pain VAS and degree of MMA	˗0.907	<0.001	˗0.942	<0.001	˗0.856	<0.001	˗0.927	<0.001
Leg pain VAS and degree of MMA	˗0.078	0.765	˗0.045	0.654	˗0.124	0.498	˗0.117	0.151
ODI and degree of MMA	˗0.407	0.035	˗0.541	<0.001	˗0.298	0.044	˗0.474	<0.001

MMA, multifidus muscle atrophy; VAS, Visual Analog Scale; ODI, Oswestry Disability Index. r represents the Spearman rank correlation coefficient.

### 3.7 Correlation analysis of the degree of lumbar spondylolisthesis with VAS scores for low back and leg pain and ODI scores

Patients in the L3, L4, and L5 groups were classified into Grade I and Grade II lumbar spondylolisthesis according to the Meyerding classification. A comparison of VAS scores for low back pain between patients with Grade I and Grade II spondylolisthesis in each group exhibited no statistically significant differences (*p* > 0.05; Tables 7–9). However, comparisons of VAS scores for leg pain and ODI scores revealed statistically significant differences (*p* < 0.05; Tables 7–9).

**Table 7:** Comparative analysis of different grades of DLS in the L3 segment with preoperative low back pain, leg pain, and ODI scores.

Variables	Grade I	Grade II	*t*	*p-*values
Preoperative VAS score for low back pain	*n* = 24	*n* = 3		
Mean ± SD	7.42 ± 0.65	7.33 ± 1.53	˗0.734	0.3400
Preoperative VAS score for leg pain	*n* = 14	*n* = 3		
Mean ± SD	6.21 ± 0.89	8.33 ± 0.58	2.703	0.0069
Preoperative ODI (%)	*n* = 24	*n* = 3		
Mean ± SD	55.83 ± 6.54	73.33 ± 5.77	2.885	0.0039

DLS, degenerative lumbar spondylolisthesis; VAS, Visual Analog Scale; ODI, Oswestry Disability Index; SD, standard deviation.

**Table 8:** Comparative analysis of different grades of DLS in the L4 segment with preoperative low back pain, leg pain, and ODI scores.

Variables	Grade I	Grade II	*t*	*p-*values
Preoperative VAS score for low back pain	*n* = 126	*n* = 14		
Mean ± SD	7.29 ± 0.93	7.21 ± 1.19	˗0.668	0.429
Preoperative VAS score for leg pain	*n* = 88	*n* = 14		
Mean ± SD	6.14 ± 0.89	7.71 ± 2.05	4.891	< 0.0001
Preoperative ODI (%)	*n* = 126	*n* = 14		
Mean ± SD	55.40 ± 8.36	72.86 ± 7.26	5.608	< 0.0001

DLS, degenerative lumbar spondylolisthesis; VAS, Visual Analog Scale; ODI, Oswestry Disability Index; SD, standard deviation.

**Table 9:** Comparative analysis of different grades of DLS in the L5 segment with preoperative low back pain, leg pain, and ODI scores.

Variables	Grade I	Grade II	*t*	*p-*values
Preoperative VAS score for low back pain	*n* = 40	*n* = 6		
Mean ± SD	7.08 ± 0.80	7.67 ± 0.82	1.601	0.109
Preoperative VAS score for leg pain	*n* = 26	*n* = 6		
Mean ± SD	6.65 ± 0.85	7.17 ± 3.06	2.388	0.017
Preoperative ODI (%)	*n* = 40	*n* = 6		
Mean ± SD	54.50 ± 8.76	75.00 ± 5.48	3.931	< 0.0001

DLS, degenerative lumbar spondylolisthesis; VAS, Visual Analog Scale; ODI, Oswestry Disability Index; SD, standard deviation.

### 3.8 Comparison of laboratory test findings between the mild and severe groups

According to the Kjaer classification, patients were categorised into the Mild and Severe groups based on the degree of MMA. Univariate analysis revealed no statistically significant differences in laboratory test indicators between the Severe and Mild groups, including red blood cell count, haematocrit levels, haemoglobin levels, platelet count, prothrombin time, activated partial thromboplastin time, international normalised ratio, total protein, albumin, globulin, and the albumin-to-globulin ratio (*p* > 0.05; Table 10).

**Table 10:** Comparison of laboratory test indicators between the mild and severe groups.

Laboratory tests	Mild group (*n* = 110)	Severe group (*n* = 103)	*p-*values
Red blood cell count (×10^12^/L, $\bar{x}\;$ ± *s*)	4.5 ± 0.4	4.4 ± 0.5	0.273
Haematocrit (%, $\bar{x\;}$ ± *s*)	40.8 ± 4.0	40.5 ± 4.2	0.467
Haemoglobin (g/L, $\bar{x\;}$ ± *s*)	135.9 ± 15.6	134.9 ± 15.5	0.504
Platelet count (×10^9^/L, $\bar{x}$ ± *s*)	220.6 ± 58.6	215.5 ± 55.5	0.392
Prothrombin time (s, $\bar{x}$ ± *s*)	11.4 ± 0.9	11.4 ± 0.8	0.694
Activated partial thromboplastin time (s, $\bar{x\;}$ ± *s*)	29.4 ± 3.1	29.3 ± 2.6	0.712
International normalised ratio ( $\bar{x\;}$ ± *s*)	1.1 ± 0.1	1.1 ± 0.1	0.683
Total protein (g/L, $\bar{x}\;$ ± *s*)	68.71 ± 2.68	68.41 ± 2.93	0.78
Albumin (g/L, $\;\bar{x}$ ± *s*)	42.77 ± 2.54	43.63 ± 2.64	0.389
Globulin (g/L, $\bar{x\;}$ ± *s*)	25.94 ± 2.64	24.78 ± 2.23	0.222
Albumin-to-globulin ratio	1.67 ± 0.24	1.78 ± 0.21	0.225

### 3.9 Comparison of basic characteristics between patients with the mild and severe groups

Univariate risk factor analysis revealed no statistically significant differences in sex, hypertension, or diabetes mellitus between the Mild and Severe groups (Kjaer classification) (*p* > 0.05). However, age, BMI, and osteoporosis revealed statistically significant differences between the two groups (*p* < 0.05; Table 11).

**Table 11:** Comparison of general characteristics between the Mild and Severe groups.

Variables	Mild group	Severe group	*p-*values
	*n* = 110	*n* = 103	
Age	61.1 ± 9.35	65.1 ± 8.73	<0.001
Male, n(%)	33(30.00)	23(22.33)	0.204
BMI(kg/m^2^)	24.47 ± 2.75	25.67 ± 3.43	0.006
Osteoporosis, n(%)	42(38.18)	65(63.11)	<0.001
Hypertension, n(%)	50(45.45)	47(45.63)	0.979
Diabetes mellitus, n(%)	15(13.64)	18(17.48)	0.439

BMI, body mass index; SD, standard deviation.

### 3.10 Multivariate logistic regression analysis of risk factors for MMA in DLS patients

Multivariate logistic regression analysis identified age (beta coefficient [B] = 1.246, odd ratio [OR] = 3.476, 95% confidence interval [CI] = 2.109–5.729), BMI (B = 0.740, OR = 2.096, 95% CI = 1.144–3.839), and osteoporosis (B = 0.041, OR = 1.042, 95% CI = 1.018–1.067) as significant risk factors for the exacerbation of MMA in DLS patients (*p* < 0.05; Table 12).

**Table 12:** Multivariate logistic regression analysis of risk factors for MMA exacerbation.

Variables	OR	95% CI	B-values	*p-*values
Age	3.476	2.109–5.729	1.246	<0.001
BMI	2.096	1.144–3.839	0.740	0.017
Osteoporosis	1.042	1.018–1.067	0.041	0.001

MMA, multifidus muscle atrophy; BMI, body mass index; CI, confidence interval; OD, odds ratio; B, B coefficient.

## 4 Discussion

The concept of MMA was first introduced by Mattila et al., who suggested a significant association with low back pain and lumbar disc herniation (19). DLS, a prevalent degenerative disease of the lumbar spine, is primarily attributed to diminished lumbar stability. This condition is characterised by low back pain due to vertebral spondylolisthesis and leg pain caused by nerve root compression. MMA is closely associated with vertebral spondylolisthesis, with patients experiencing low back pain exhibiting significant fat infiltration and multifidus muscle atrophy compared to healthy individuals (20). MRI of the lumbar spine frequently reveals pronounced MMA at the level of spondylolisthesis in DLS patients (21). Previous studies have demonstrated a correlation between MMA and facet joint hypertrophy, spinal stenosis, and lumbar spondylolisthesis (22).

To minimise the potential influence of low back pain on MMA, we recruited 20 healthy older adult volunteers as a control group. We compared the degree of MMA between DLS patients and the control group and found that MMA at the L3/4, L4/5, and L5/S1 segments was significantly more pronounced in DLS patients compared to the control group. This finding indicates that the multifidus muscle undergoes substantial atrophy in DLS patients. Furthermore, we analysed whether the degree of MMA differed between spondylolisthesis and non-spondylolisthesis segments across the three groups. Our findings demonstrated a significant increase in MMA in spondylolisthesis segments, confirming that these segments experience more severe atrophy. In addition, this deterioration appears to influence adjacent lumbar segments, exacerbating MMA progression. Previous studies have reported similar findings in patients with degenerative lumbar stenosis, demonstrating that MMA is more severe in affected segments compared to adjacent unaffected segments (23–25). These findings are consistent with our findings, which indicate that MMA increases with age and is significantly more pronounced in DLS patients, particularly in spondylolisthesis segments. Moreover, our findings revealed a positive correlation between MMA in spondylolisthesis and non-spondylolisthesis segments of the lumbar spine, indicating a broader impact of muscle atrophy beyond the directly affected regions.

In DLS, excessive motion in adjacent segments increases stress on the paravertebral muscles, leading to direct injury, particularly affecting the multifidus muscle. In addition, this process compromises the nerve supply to the paravertebral muscles, including the multifidus. The nerve branches pass through the fibrous tunnel formed by the ligamentum flavum, and lumbar spondylolisthesis, along with facet joint hypertrophy, exacerbate local muscle and nerve damage (26).

VAS and ODI scores are commonly used to evaluate low back pain severity and lumbar function in spinal disorders (27). In patients with degenerative lumbar scoliosis, MMA exhibits a significant positive correlation with VAS scores for low back pain and ODI scores for lumbar function, indicating that MMA significantly contributes to a decline in quality of life (28). In addition, improving MMA can lead to a significant reduction in VAS and ODI scores, enhancing patients’ quality of life. Several studies analysing the effects of unilateral multi-segmental minimally invasive interbody fusion on paravertebral muscles have found that a reduction in the LCSA is closely associated with postoperative VAS scores for low back pain (29, 30). However, no significant correlation has been observed between LCSA reduction and VAS scores for leg pain (31). These findings indicate that MMA is significantly associated with degenerative lumbar diseases and plays a crucial role in low back pain and lumbar dysfunction in DLS patients. Therefore, during lumbar spine surgery, surgeons need to utilise minimally invasive techniques that maximise the protection and preservation of multifidus muscle to reduce residual postoperative lower back pain.

Moreover, we analysed the correlation between the degree of MMA and the severity of DLS with low back pain and lumbar dysfunction. Our findings revealed that all three groups of spondylolisthesis patients experienced varying degrees of low back pain and referred pain in the lower limbs. A positive correlation was observed between the degree of MMA and low back pain and lumbar dysfunction; however, no correlation was observed with leg pain. Furthermore, when comparing Grade II spondylolisthesis to Grade I spondylolisthesis, no statistically significant differences were observed in VAS scores for low back pain. Conversely, statistically significant differences were observed in VAS scores for leg pain and dysfunction.

The causal relationship between DLS and MMA remains uncertain. It is widely believed that DLS leads to compression of the medial branch of the segmental nerve, which innervates and nourishes the multifidus muscle, resulting in denervation and subsequent atrophy (32). In DLS patients, the severity of MMA is closely associated with the degree of spondylolisthesis and is thought to contribute to disease progression (15). Several studies have demonstrated a positive correlation between the extent of MMA and the severity of lumbar spondylolisthesis in DLS patients (33, 34). Similar findings have been reported in patients with degenerative lumbar stenosis, where MMA is more pronounced in the degenerated segment than in adjacent normal segments (23). Guo et al. analysed risk factors for spondylolisthesis in 90 DLS patients and suggested that MMA contributes to lumbar instability, increasing the likelihood of vertebral slippage (35). However, the extent of MMA’s contribution relative to DLS among various risk factors remains unclear. MMA is considered a causative factor in spondylolisthesis, as it progressively worsens with age, leading to chronic low back pain, reduced spinal tension, and diminished control over external loads, resulting in lumbar instability (18). Several studies have suggested that DLS may contribute to MMA, suggesting that, in the early stages of lumbar spondylolisthesis, the paravertebral muscles compensate through hypertrophy to maintain lumbar stability. However, as the condition advances, these muscles may undergo atrophy due to decompensation (36, 37).

Therefore, the causal relationship between MMA and DLS remains unclear. Previous studies on their correlation indicate a lack of direct evidence to definitively establish a causal link between the two.

The precise etiological factors contributing to the development of MMA remain uncertain, impeding the implementation of effective early preventive strategies for DLS through MMA-targeted interventions. Consequently, the identification and mitigation of high-risk factors for MMA in DLS patients are of paramount clinical significance. We stratified patients into Mild and Severe MMA groups based on the Kjaer classification system (15) and conducted a retrospective analysis to delineate the independent risk factors for MMA in DLS. Our findings reveal that advanced age, elevated BMI, and osteoporosis are independently associated with the exacerbation of MMA in DLS patients.

Previous studies have established muscle atrophy as a primary determinant of diminished muscle strength, precipitating a cascade of functional imbalances within the musculoskeletal system (38–41). Notably, multifidus muscle quality declines progressively, with an estimated 1% annual reduction after the age of 40 (12, 13). Degenerative changes and fat infiltration in the multifidus muscle contribute to weakened voluntary contraction, leading to muscle atrophy and, ultimately, lumbar instability (14). These findings suggest that age is a significant contributor to MMA.

Comparative analyses of paravertebral muscle morphology between patients with lumbar spinal stenosis and asymptomatic middle-aged and older adult individuals have demonstrated age as a significant independent predictor of paravertebral muscle atrophy. Notably, the impact of age on muscle atrophy appears to be more pronounced in patients with lumbar spinal stenosis (42). Furthermore, studies investigating the correlation between back muscle characteristics and tenderness sensitivity in patients with low back pain have identified age as a critical factor contributing to fat infiltration within the multifidus muscle, leading to its atrophy (43).

MMA is characterised by an increase in fat infiltration. BMI, a well-established indicator of obesity, reflects a higher proportion of adipose tissue, which can contribute to increased fat infiltration in the muscles. We identified BMI as an independent risk factor for the exacerbation of MMA in DLS patients. Notably, patients in the Severe MMA group exhibited a higher BMI, suggesting that elevated BMI is linked to increased fat infiltration and more severe MMA.

Furthermore, a recent study revealed that higher BMI and vitamin D deficiency are significant risk factors for paravertebral muscle atrophy and low back pain in postmenopausal women, with both factors exerting a cumulative effect that worsens muscle atrophy and pain severity (44). In addition, Ogon et al. used MRI to analyse the factors influencing intracellular and extracellular lipid accumulation in multifidus fat infiltration among patients with chronic low back pain. Their findings indicate that higher BMI is associated with increased extracellular lipid infiltration, exacerbating MMA (45).

We identified osteoporosis as an independent risk factor for the progression of MMA in DLS patients. The presence of osteoporosis indicates a metabolic imbalance in the musculoskeletal system, which significantly increases the risk of MMA.

Lee et al. reported a significant association between multifidus fat infiltration and osteoporotic vertebral compression fractures. In addition, paravertebral muscle atrophy has been associated with the recurrence of acute osteoporotic vertebral compression fractures in patients following percutaneous vertebroplasty, emphasising the connection between muscle atrophy and osteoporosis-related fractures (46). In older women, both decreased bone density and paravertebral muscle atrophy are prevalent. Notably, individuals with reduced bone density have a 5.7-fold higher likelihood of developing paravertebral muscle atrophy, emphasising a significant association between bone loss and muscle degeneration (47, 48).

## 5 Conclusion

DLS patients experience MMA, which is more pronounced at the spondylolisthesis segment. The severity of MMA exhibits a positive correlation with VAS scores for low back pain and ODI, but is not associated with VAS scores for leg pain. Compared to Grade I lumbar spondylolisthesis, Grade II does not show a significant difference in MMA severity or low back pain VAS scores; however, patients experience more severe leg pain and lumbar dysfunction. In addition, age, BMI, and osteoporosis are independent risk factors for MMA progression in DLS patients. Clinically, patients with these risk factors require careful monitoring, and effective prevention and treatment strategies should be actively implemented, including back muscle strengthening exercises, weight management, and anti-osteoporosis therapy.

## Data Availability

The original contributions presented in the study are included in the article/supplementary material, further inquiries can be directed to the corresponding author.
